# Transcriptome analysis of *Pseudomonas aeruginosa* PAO1 grown at both body and elevated temperatures

**DOI:** 10.7717/peerj.2223

**Published:** 2016-07-19

**Authors:** Kok-Gan Chan, Kumutha Priya, Chien-Yi Chang, Ahmad Yamin Abdul Rahman, Kok Keng Tee, Wai-Fong Yin

**Affiliations:** 1 ISB (Genetics & Molecular Biology), Faculty of Science, University of Malaya, Kuala Lumpur, Malaysia; 2 School of Life Sciences, Heriot-Watt University, Edinburgh, United Kingdom; 3 BioEasy Sdn Bhd, Shah Alam, Selangor, Malaysia; 4 Department of Medical Microbiology, Faculty of Medicine, University of Malaya, Kuala Lumpur, Malaysia

**Keywords:** Heat shock, RNA sequencing, Pseudomonas aeruginosa PAO1, Transcriptome, Gene expression

## Abstract

Functional genomics research can give us valuable insights into bacterial gene function. RNA Sequencing (RNA-seq) can generate information on transcript abundance in bacteria following abiotic stress treatments. In this study, we used the RNA-seq technique to study the transcriptomes of the opportunistic nosocomial pathogen *Pseudomonas aeruginosa* PAO1 following heat shock. Samples were grown at both the human body temperature (37 °C) and an arbitrarily-selected temperature of 46 °C. In this work using RNA-seq, we identified 133 genes that are differentially expressed at 46 °C compared to the human body temperature. Our work identifies some key *P. aeruginosa* PAO1 genes whose products have importance in both environmental adaptation as well as in vivo infection in febrile hosts. More importantly, our transcriptomic results show that many genes are only expressed when subjected to heat shock. Because the RNA-seq can generate high throughput gene expression profiles, our work reveals many unanticipated genes with further work to be done exploring such genes products.

## Introduction

*Pseudomonas aeruginosa* is an opportunistic nosocomial pathogen which is a threat to public health (Lyczak, Cannon & Pier, 2000). Its success as a pathogen is due to being well adapted to changes in environmental factors (Cheng et al., 2014; Livermore, 2002). Due to this versatility, its virulence determinants and host factors *P. aeruginosa* is the known causal agent in a myriad of human diseases (Cheng et al., 2014).

*P. aeruginosa* has previously been shown to thrive in hostile environments and has a rapid response and adaptation to abiotic stresses such as elevated and reduced temperature (Farrell & Rose, 1968; Schurr et al., 1995). This rapid adaptation of *P. aeruginosa* is accompanied by changes in its genomic regulatory network modulating the global expression and activities of genes essential for their survival (Burrowes et al., 2006; Schuster et al., 2003). In one of the earliest studies conducted on heat shock response of *P. aeruginosa,* it was observed that the syntheses of 17 proteins was enhanced following the transfer of cells from 30 to 45 °C (Allan et al., 1988). It was later discovered that the principle sigma factor, σ, encoded by *rpoDA*, was among the proteins expressed upon elevation of temperature from 30 to 42 °C (Fujita et al., 1993). Furthermore, the synthesis of *rpoH* and *groEL* mRNA are also induced following heat shock, suggesting that the transcription of these genes is regulated by heat shock RNA polymerases as well as by the principle RNA polymerase. The findings were further supported by the discovery of sequences for the polymerases in the upstream promoter region of the genes (Fujita et al., 1993; Fujita, Amemura & Aramaki, 1998). When bacterial cells are exposed to elevated temperatures, the transcription shifts from σ^70^ to σ^32^. This enables the RNA polymerases to recognise the heat shock genes which are crucial for its adaptation in the drastic change in the surrounding environment (Straus, Walter & Gross, 1990).

A more recent study carried out on *P. aeruginosa* isolated from cystic fibrosis (CF) patients small heat shock proteins (sHSPs), namely Hp25 and Hp18, were discovered. These sHSPs were highly expressed under both standard laboratory conditions and conditions that mimicked the sputum-like environment of the CF patients. The authors suggest that the discovery of the sHSPs in both conditions was due to the proteins acting as molecular chaperones helping with the adaptability of *P. aeruginosa* to diverse environments (Sriramulu, 2009). Under stressful conditions, these sHSPs interact with affected proteins, preventing their aggregation, with the process continuing with the aid of chaperone proteins (Wang & Spector, 2000).

In order to understand the heat shock response of *Pseudomonas*, several studies on other species in the *Pseudomonas* genus have been carried out. A study conducted on *Pseudomonas syringae* shows that the expression of *dnaK* increased significantly when cells that were initially incubated at 18 °C were transferred to 35 °C. However, the results indicated that although *P. syringae* responded by producing DnaK, it did not help in adjusting to the gradual change to an elevated temperature (Keith, Partridge & Bender, 1999). In another study in *Pseudomonas putida* KT2442, the role of several molecular chaperones, namely ClpB, DnaJ, CbpA, and DjlA, were elucidated. The increase in the expression heat shock proteins (Hsps) mentioned was not significant when transferred from 30 to 33 °C. However, when shifted to 35 °C the expression of the DnaJ, GroEL, HtpG, and ClpB was increased. At larger temperature shifts up to 42 °C, expression of these Hsps was increased further. At a more elevated temperature of 45 °C, expression of DnaK, GroEL, and HtpG increased only during the first 10 min while the expression of ClpB continued to increase (Ito et al., 2014).

Next Generation sequencing (NGS) has been proven to be an invaluable technology to study bacteria genomes (Chan, Yin & Lim, 2014; Chan et al., 2013; Chan et al., 2012; Forde et al., 2014; Lau, Yin & Chan, 2014; Tan, Yin & Chan, 2014). In particular RNA-seq can provide a complete coverage of protein-coding genes, intergenic regions and non-coding RNA and small, regulatory RNA populations in a given genome (Wang, Gerstein & Snyder, 2009). Over the past decade, the heat stress responses of other *Pseudomonas* species have been extensively studied. Not much work has been done to investigate the transcriptional response of *P. aeruginosa* PAO1 to elevated temperature using NGS methodologies. To address this, we have used the RNA-seq technique to look at changes in the transcriptome of *P. aeruginosa* PAO1 exposed to heat shock. Previous heat shock studies have shown that a temperature difference of at least 15 °C between the normal and elevated growth temperature provides the best information (Ito et al., 2014). With reference to that study, we chose to grow *P. aeruginosa* PAO1 at 37 and 46 °C.

## Materials and Methods

### Culture conditions and growth study

Growth of *P. aeruginosa* PAO1 was determined at 37 and 46 °C. Briefly, *P. aeruginosa* PAO1 was cultured for 18 h at 37 °C. The overnight grown culture was adjusted to OD_600_ of 0.01. Aliquots of cells (200 μL) were then dispensed into wells of a sterile 96-well microtitre plate (Priya, Yin & Chan, 2013; Tan, Yin & Chan, 2013) and incubated at 37 and 46 °C, respectively. The OD_600_ was measured at intervals of 4 h for 24 h using a microplate reader (Tecan Infinite M200, Mannerdorf, Switzerland). The growth of *P. aeruginosa* at 37 and 46 °C was also determined by looking at its colony forming unit (CFU). The CFU count at the stipulated temperature was determined at desired time points. Briefly, 100 μL of the cultures grown at 37 and 46 °C were serially diluted and 100 μL diluted cultures were plated on Luria-Bertani Agar (LB). The plates were incubated at 37 °C for 18 h before the determination of CFU per mL.

### Heat shock treatment of *P. aeruginosa* PAO1

*P. aeruginosa* PAO1 cells were taken from −80 °C stock cultures and grown on LB agar in order to obtain pure colonies. These were subsequently inoculated into fresh sterile LB broth for 18 h at 37 °C with shaking at 220 rpm. Overnight seed cultures were then sub-cultured (1 mL) into 100 mL of fresh, sterile LB broth and grown to mid-exponential phase (OD_600_ = 0.5) at 37 °C. The 10 mL of culture was then transferred into sterile tube (50 mL volume tube) pre-warmed at 37 and 46 °C, respectively, and immediately exposed to heat shock for 30 min (with shaking) by incubating in water baths pre-heated to 37 and 46 °C, respectively. This was followed immediately by RNA extraction. Experiments were performed in triplicate.

### RNA extraction and cDNA synthesis

Total RNA was extracted using MasterPure™ RNA Purification Kit (Epicentre, WI, USA) as per the manufacturer’s instructions. Precipitated RNA samples were resuspended in sterile RNase-free water. Purity of RNA samples was assessed using a NanoDrop 2000 Spectrophotometer (Thermo Scientific, MA, USA) and only samples with A_260_/A_280_ and A_260_/A_230_ values > 2.0 were chosen for further work. The quality of the extracted RNA samples was also determined using an Agilent Bioanalyzer-RNA 6000 Pico Kit (Agilent Technologies, CA, USA). RNA samples with RNA Integrity Numbers (RIN) of value > 7.5 were chosen for rRNA depletion using Ribo-Zero™ rRNA Removal Kits (Bacteria) (Epicentre, WI, USA) prior to cDNA synthesis. Samples were then checked for the loss of intact rRNA using Agilent Bioanalyzer. Synthesis of cDNA was performed using the protocols of ScriptSeq™ v2 RNA-seq Library Preparation Kit (Epicentre, WI, USA). The quality of the RNA-seq cDNA library was confirmed using Agilent Bioanalyzer-High Sensitivity DNA Chip.

### cDNA library preparation and RNA-seq

Quantification of the RNA-seq transcriptome library was performed using a Qubit® dsDNA High Sensitivity (HS) Assay Kit (Life Technologies, CA, USA) and normalised to a concentration of 4 nM. Normalised samples were denatured with 0.2 N NaOH and diluted 20 pM using pre-chilled Hybridisation Buffer (HT1) (Illumina, CA, USA). The 20 pM transcriptome libraries were further diluted to 10 pM with pre-chilled HT1 buffer and combined with 1% denatured and diluted PhiX control prior to sequencing using MiSeq platform.

All resulting nucleotide sequence accession number is available in public databases. The DNA sequences from this transcriptomics project has been deposited at Sequence Read Archive (NCBI/SRA) under the accession number SRP066875. The transcriptome data have been deposited in BioProject in GenBank via Bioproject number PRJNA304652.

### Validation of RNA-seq using real time-PCR (RT-PCR)

RT-PCR was performed to quantify and validate the level of *P. aeruginosa* PAO1 gene expression that were affected when exposed to an elevated temperature. *P. aeruginosa* PAO1 cells were subjected to heat shock and their RNA was extracted once again as an independent experiment to determine the reproducibility of the data. One microgram of RNA was reverse transcribed into cDNA using the QuantiTect Reverse Transcription Kit (Qiagen, USA). For the quantitative RT-PCR, the amplification was performed using the KAPA SYBR® FAST qPCR Kit Master Mix Universal (Kapa Biosystems, USA) on the Bio-Rad CFX96 real-time system (Bio-Rad, CA, USA). Genes from the upregulated and downregulated gene list obtained from RNA-seq result were selected and the primers for the genes were designed using Primer 3 version 0.4.0 (http://bioinfo.ut.ee/primer3-0.4.0/). The RT-PCR condition used was as follows: initial denaturation at 95 °C for 3 min, followed by a 40 cycles of denaturation at 95 °C for 3 s and annealing/extension at 55.7 °C for 30 s. The fluorescent signals were quantified at the end of each cycle. Data obtained were analysed using the Bio-Rad CFX Manager™ Software version 1.6. Reference genes with expression stability values (*M* value) of less than 0.7 were selected as reference genes for normalisation. The selected reference genes were *cheZ, proC, recA, rpoB, gyrB, oprL,* and *plsY* (Matthijs et al., 2013; Savli et al., 2003). Upregulated genes: *clpB, dnaJ, dnaK* and *grpE* and downregulated genes: *tssG1*, *hsiC2* and *pilA* were selected.

### Differential expression analysis

RNA-seq read quality assessment was done using FastQC (version 0.11.2) (Andrews, 2010; Babraham Bioinformatics, Cambridge, UK). The dataset was analyzed using tuxedo suite protocol for differential gene expression analysis (Trapnell et al., 2012). Tophat (version 2.0.11) was used to align the paired end reads against *P. aeruginosa* PAO1 reference genome (GenBank accession number AE004091). Cuffdiff2 (version 2.2.0), one of the subprograms in cufflinks package was used for differential expression purpose (*p* corrected value/*q* ≤ 0.05) [18]. Fold change of the expression profile was measured using log_2_ FPKM (Fragments per Kilobase of transcript per Million mapped reads). For QC and generation of plots expression, we used Volcano, Box, Scv, Density, Scatter plot, PCA and heat map as available in the R package cummeRbund (version 2.6.1) based on output from cuffdiff2. For RNA-seq analysis, the total number of reads per gene between samples was normalized using FPKM (Trapnell et al., 2010).

## Results

### Survival studies

**Figure 1:**
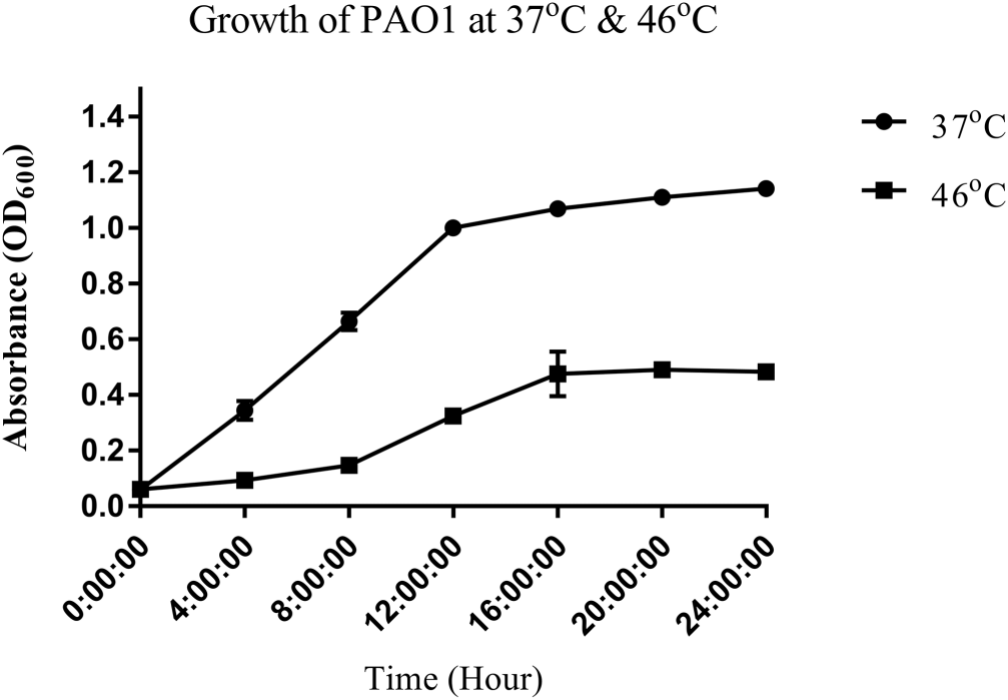
Growth curve of *P. aeruginosa* PAO1 incubated at 37 °C (circle) and 46 °C (square).

To study the heat shock response of the nosocomial pathogen *P. aeruginosa* PAO1, we first determined the growth of *P. aeruginosa* PAO1 at 37 and 46 °C (Fig. 1). Growth curve studies revealed that *P. aeruginosa* PAO1 could adapt to temperature at 46 °C (Fig. 1). Following a 30 min heat shock, the CFU count for *P. aeruginosa* PAO1 at 37 and 46 °C were 1.37 × 10^8^ and 1.33 × 10^8^, respectively. The growth rate of *P. aeruginosa* PAO1 grown at 37 and 46 °C declined over 24 h and it appears that 46 °C has adverse effect on the growth of *P. aeruginosa* PAO1 (Table 1).

**Table 1:** *P. aeruginosa* PAO1 cells viability at 37 and 46 °C.

Time (h)	37 °C (CFU/mL)	46 °C (CFU/mL)
6	7.43 ± 0.78 × 10^6^	7.90 ± 0.71 × 10^4^
12	1.32 ± 0.08 × 10^9^	1.08 ± 0.11 × 10^5^
18	1.76 ± 0.05 × 10^9^	2.03 ± 0.05 × 10^5^
24	2.19 ± 0.17 × 10^9^	2.52 ± 0.28 × 10^5^

**Figure 2:**
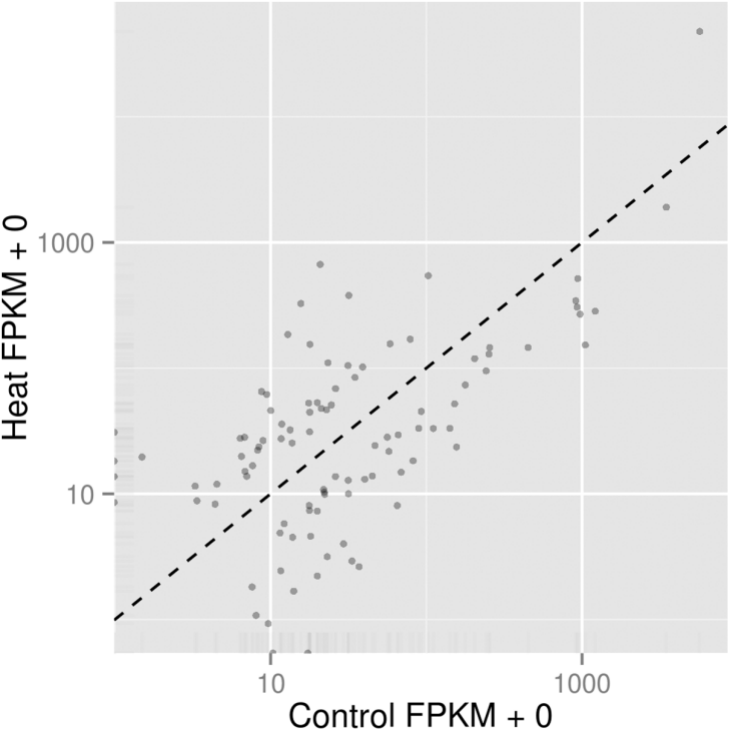
Comparison of significant gene expression between *P. aeruginosa* PAO1 samples with (Heat) and without heat shock (Control). The significant level whether it is highly similar is made based on figures generated with *p* corrected value ≤ 0.05.

In order to understand the cellular response of *P. aeruginosa* PAO1 to heat shock, we utilised an RNA-seq approach to study transcriptome changes. Experiments were carried out in triplicate with cells grown at 37 °C followed by heat shock at 46 °C for 30 min. More than 90% of all trimmed RNA-seq reads aligned to coding regions of the *P. aeruginosa* genome (Table 2). Overall expression levels in the triplicate samples of both control and heat shock samples were similar to each other (Fig. 2). The significant level whether it is highly similar is made based on figures generated with *p* corrected value ≤ 0.05. The RNA-seq data obtained therefore have sufficient quality for further transcriptome analysis.

**Table 2:** Summary of illumina RNA-seq data. *P. aeruginosa* PAO1 cells grown at 37 °C (Control) and exposed to 46 °C for 30 min (Heat), numbers (_1, _2, _3) following “Control” and “Heat” represent replicate experiment in triplicate.

Label	Total reads	Overall read mapping rate
Control_1	5,220,526	91.00%
Control_2	4,203,880	91.30%
Control_3	2,989,148	92.20%
Heat_1	4,537,188	89.90%
Heat_2	4,613,056	90.20%
Heat_3	7,639,516	90.80%

**Figure 3:**
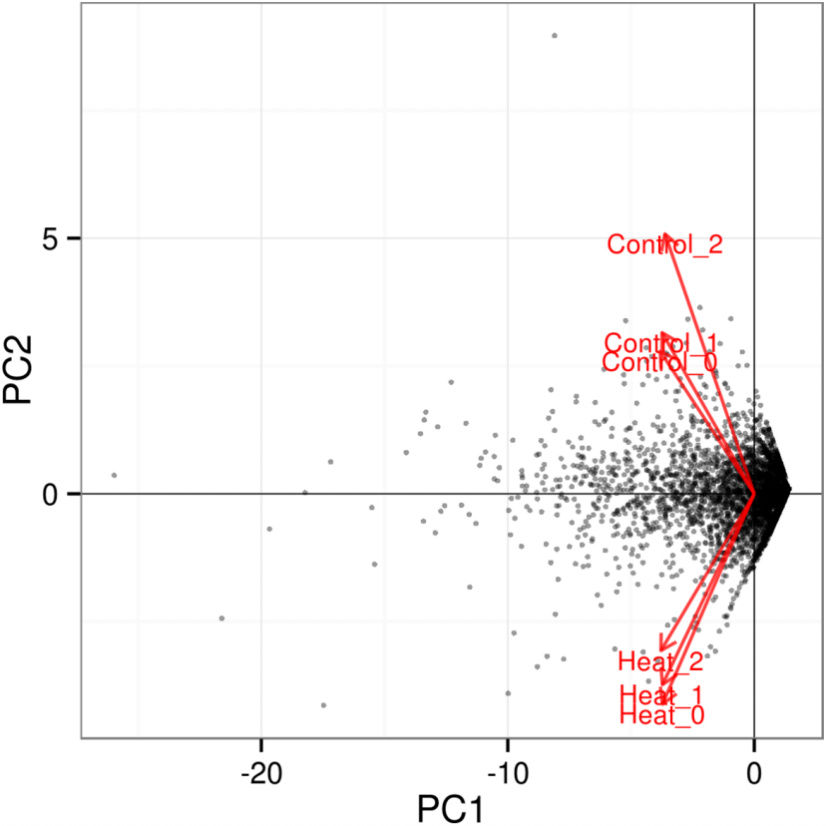
PCA plot for triplicates of each heat treatment. All three samples of each heat treatment, 37 and 46 °C are clustered together, indicating good replication among samples. The significant level whether it is a clear profile separation is made based on figures generated with *p* corrected value ≤ 0.05.

Further analysis among the triplicates of each heat treatment experiments at 37 and 46 °C showed that the RNA-seq datasets from similar treatments clustered together, as depicted in the PCA plot (Fig. 3). The significant level whether it is clear profile separation is made based on figures generated with *p* corrected value ≤ 0.05. This indicates that experimental replication was good and there was little variation among the triplicates of the same treatment.

**Figure 4:**
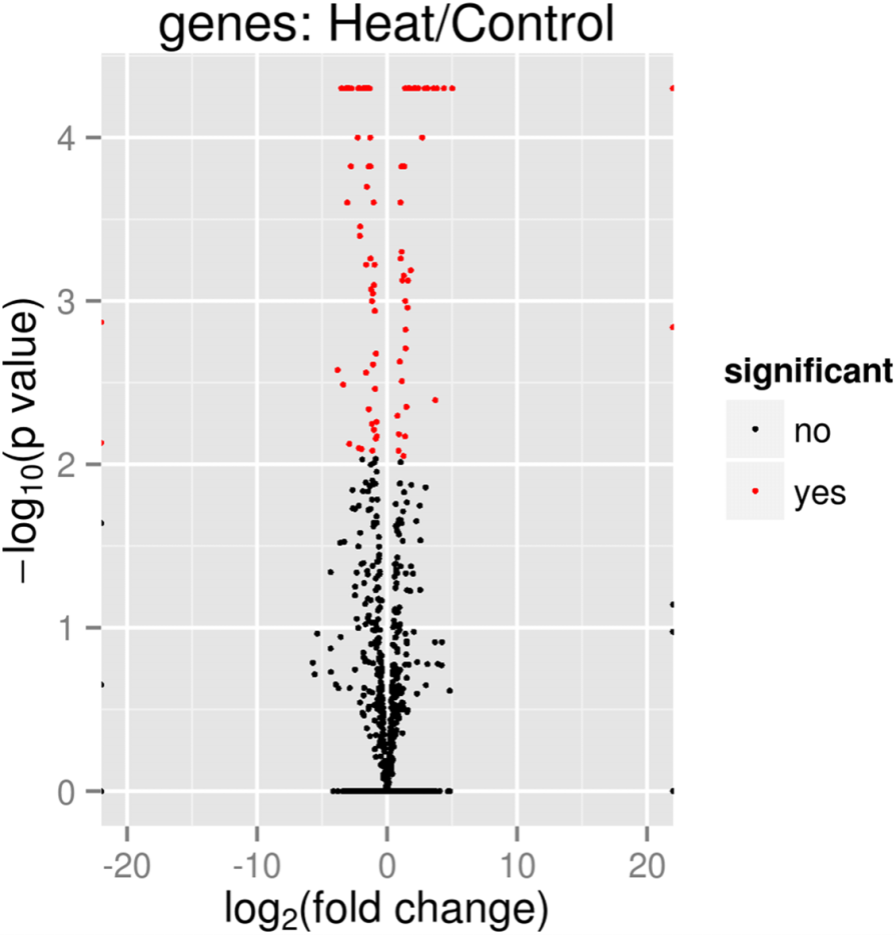
Volcano plot of *P. aeruginosa* PAO1 gene expression pattern. In the volcano plot, the red dot shows significant genes below or at alpha = 0.05; Control: exposure to 37 °C; Heat: exposure to 46 °C.

In total 133 genes were significantly differentially expressed when *P. aeruginosa* PAO1 cells were grown under heat shock at 46 °C (Data S1). Tables 3 and 4 show the list of the significant up- and downregulated genes with a cut-off value of *q* < 0.05. The volcano plot (Fig. 4) indicates the genes affected by the heat shock at 46 °C. The red dots in the volcano plot represents the 133 significant genes (*q* ≤ 0.05) while the black dots represent 5,545 non-significant genes.

**Table 3:** *P. aeruginosa* PAO1 genes significantly upregulated when exposed to heat shock. The below table shows the list of the significant upregulated genes with a cut-off value of *q* < 0.05.

Gene ID	Gene name	Value 1	Value 2	log_2_ (fold change)	Test statistics	*p* value	*q* value
gene4634	*clpB*	21.5321	641.183	4.89618	10.3745	5e-05	0.00069884
gene791	*asrA*	16.2477	308.424	4.24661	8.52567	5e-05	0.00069884
gene4562	PA4474	1.57511	19.704	3.64497	3.83695	0.00325	0.0224511
gene4873	*dnaK*	31.4943	360.251	3.51584	7.06391	5e-05	0.00069884
gene4874	*grpE*	16.8391	184.341	3.45249	5.52579	5e-05	0.00069884
gene583	*dnaG*	4.13693	35.1505	3.08691	5.14367	5e-05	0.00069884
gene4872	*dnaJ*	18.0378	149.49	3.05095	5.85741	5e-05	0.00069884
gene4983	PA4870	6.29367	50.96	3.01739	2.55899	0.0056	0.0323615
gene1625	*htpG*	8.62707	61.2625	2.82806	5.19305	5e-05	0.00069884
gene4871	*dapB*	1.20712	8.33066	2.78687	1.4514	0.00925	0.0448327
gene920	*rsmA*	3626.1	22,913.4	2.6597	5.64019	5e-05	0.00069884
gene5125	*waaG*	1.80622	10.3941	2.52472	2.08787	0.0063	0.0346941
gene1439	PA1414	8.59171	47.2851	2.46037	2.24087	0.00385	0.0247286
gene5168	*hslV*	6.10316	32.0818	2.39413	2.24884	0.00475	0.0282649
gene4470	*groEL*	100.754	519.342	2.36584	4.78994	5e-05	0.00069884
gene582	*rpoD*	23.8147	118.195	2.31124	4.5964	5e-05	0.00069884
gene4132	PA4061	8.92965	42.6481	2.25581	3.53421	5e-05	0.00069884
gene3889	PA3819	12.7016	59.714	2.23305	3.08932	0.0001	0.00130652
gene5058	PA4943	6.4703	27.5441	2.08984	3.39868	5e-05	0.00069884
gene775	*mucA*	31.219	126.381	2.01728	3.61801	5e-05	0.00069884
gene3881	*hscB*	6.52321	25.7193	1.9792	2.02124	0.00635	0.0346941
gene5169	*hslU*	6.96398	26.8164	1.94513	3.28447	5e-05	0.00069884
gene2624	*gacA*	6.74877	21.7267	1.68678	2.04244	0.00395	0.0247286
gene672	*tyrZ*	12.4244	39.5589	1.67082	2.95304	5e-05	0.00069884
gene1839	*lon*	32.829	101.139	1.6233	3.26832	5e-05	0.00069884
gene1803	*cmaX*	3.33028	10.2239	1.61823	1.72921	0.00745	0.0385256
gene5020	PA4907	9.76191	26.8597	1.46021	2.09157	0.0021	0.0159759
gene5057	*hflK*	17.8262	48.4164	1.4415	2.40358	0.00015	0.001803
gene3216	PA3157	9.87412	26.4346	1.4207	2.17387	0.00065	0.00630081
gene1838	*clpX*	55.3485	145.186	1.39129	2.83085	5e-05	0.00069884
gene1119	*fleQ*	4.1866	10.8754	1.37722	2.0293	0.00275	0.0196756
gene379	*rpoH*	16.6811	41.6596	1.32043	2.35423	0.00015	0.001803
gene1202	*oprH*	40.6783	99.4149	1.2892	2.48325	5e-05	0.00069884
gene846	PA0833	8.50343	20.6183	1.27781	1.71387	0.00785	0.0396458
gene5120	PA5005	5.17934	12.4491	1.2652	1.94415	0.0015	0.01202
gene4832	*cbrB*	3.4025	8.14844	1.25993	1.6656	0.0091	0.0444642
gene770	PA0758	8.55784	20.1953	1.2387	1.80381	0.0042	0.0257571
gene2622	*pgsA*	12.2261	28.4189	1.21689	1.6504	0.01045	0.049066
gene3062	*topA*	8.7912	20.359	1.21153	2.36625	5e-05	0.00069884
gene1075	PA1053	41.8306	93.3374	1.15789	2.02782	0.00105	0.00970846
gene2772	PA2735	30.8456	66.7609	1.11394	2.22459	0.0002	0.00231154
gene5358	*rho*	12.316	26.3594	1.09778	2.00644	0.00085	0.00810873
gene4882	*lldP*	4.00184	8.55432	1.09599	1.60525	0.00815	0.0408179
gene5562	PA5436	7.08683	14.5432	1.03713	1.71082	0.00505	0.0294665
gene3027	*rne*	24.3393	49.8051	1.03301	2.10105	0.00025	0.00268304
gene3884	*iscS*	24.603	48.5117	0.9795	1.92781	0.00175	0.0136591
gene4784	*hitA*	10.4753	20.2269	0.949286	1.53913	0.00885	0.0435971
gene727	PA0716	82.3112	157.32	0.934543	1.88924	0.0009	0.00845156
gene1554	*zipA*	24.8322	45.7464	0.881446	1.65677	0.0039	0.0247286
gene3946	*narG*	4.40154	8.07275	0.875052	1.61398	0.0061	0.0345435
gene4349	*nusG*	33.143	60.2758	0.862876	1.51907	0.01085	0.0497775
gene5050	*rnr*	7.88061	13.4832	0.774781	1.48185	0.01055	0.0491516
gene4862	*ftsH*	18.7479	30.9454	0.722993	1.46642	0.00975	0.046506
gene2520	PA2486	0	14.7402	inf	-nan	0.00145	0.0119377
gene4941	PA4828	0	12.6143	inf	-nan	5e-05	0.00069884

**Note:**

inf, not define; -nan, not available.

**Table 4:** *P. aeruginosa* PAO1 genes significantly downregulated when exposed to heat shock. The below table shows the list of the significant downregulated genes with a cut-off value of *q* < 0.05.

Gene ID	Gene name	Value 1	Value 2	log_2_ (fold change)	Test statistics	*p* value	*q* value
gene1688	*hsiC2*	22.6564	0.992143	−4.51323	−4.66897	0.0091	0.0463781
gene1117	PA1095	129.204	7.21324	−4.16286	−3.14279	0.00265	0.0191886
gene4346	PA4272.1	890.17	76.9649	−3.53181	−3.9605	5.00E-05	0.00069884
gene3798	PA3729	35.579	3.10911	−3.51645	−5.52012	5.00E-05	0.00069884
gene1686	*hsiA2*	19.5494	2.11828	−3.20616	−3.53665	0.0001	0.00130652
gene3555	*tli5*	72.136	8.88402	−3.02143	−5.06997	5.00E-05	0.00069884
gene5286	*arcD*	399.882	49.9991	−2.9996	−4.93225	5.00E-05	0.00069884
gene2724	*vgrG4*	13.6866	1.84111	−2.89412	−3.80312	5.00E-05	0.00069884
gene96	PA0095	8.16707	1.10164	−2.89017	−2.76659	0.00135	0.0114275
gene982	*dps*	142.736	19.455	−2.87514	−4.35522	5.00E-05	0.00069884
gene4614	*pilA*	49.0763	6.75242	−2.86155	−2.53313	0.01025	0.0485059
gene85	*tssC1*	24.7043	3.69094	−2.7427	−4.03767	5.00E-05	0.00069884
gene3101	*rmf*	78.8232	12.3991	−2.66838	−1.41941	0.0094	0.0451952
gene100	PA0099	29.7865	4.74403	−2.65048	−3.77489	5.00E-05	0.00069884
gene5140	*metY*	10.9322	1.89052	−2.53173	−2.42142	0.00025	0.00268304
gene4330	*rplV*	73.7324	13.723	−2.4257	−2.23755	0.0036	0.0243101
gene568	PA0563	74.2912	14.1381	−2.39361	−2.52297	0.00425	0.0258005
gene4663	PA4571	13.6487	2.62789	−2.37679	−3.4702	5.00E-05	0.00069884
gene4324	*rplX*	126.547	24.3781	−2.37602	−2.89574	0.0001	0.00130652
gene4331	*rpsS*	993.38	192.891	−2.36456	−3.71984	5.00E-05	0.00069884
gene5030	PA4917	16.1805	3.2644	−2.30937	−1.6795	0.0033	0.0225375
gene1784	*cysB*	63.673	13.7047	−2.21601	−3.32536	5.00E-05	0.00069884
gene3692	PA3623	14.5214	3.13913	−2.20975	−2.07658	0.0063	0.0346941
gene4615	*pilB*	7.14771	1.56705	−2.18943	−2.087	0.0085	0.042219
gene5048	*rpsF*	48.1814	10.7881	−2.15903	−2.29553	0.00685	0.0367576
gene90	*tssG1*	2.89407	0.709339	−2.02855	−2.53313	0.01025	0.0485059
gene1091	PA1069	7.53594	1.91221	−1.97854	−2.60782	0.00025	0.00268304
gene4828	*dksA*	933.27	242.762	−1.94275	−3.83989	5.00E-05	0.00069884
gene1394	PA1369	104.664	30.1285	−1.79656	−3.47606	5.00E-05	0.00069884
gene4551	PA4463	768.625	225.296	−1.77046	−3.50993	5.00E-05	0.00069884
gene1586	*ccoN2*	14.3183	4.55088	−1.65364	−2.45509	0.00045	0.0045839
gene1583	*ccoP2*	16.7355	5.37344	−1.63899	−2.09732	0.0037	0.0247078
gene5683	*atpH*	37.3157	12.1271	−1.62154	−2.24178	0.0014	0.0116861
gene411	*pilG*	43.3645	14.2223	−1.60836	−1.88132	0.0048	0.0282824
gene5288	*arcB*	458.603	150.822	−1.6044	−3.12551	5.00E-05	0.00069884
gene1004	*pyoS5*	52.8453	17.5083	−1.59374	−3.17122	5.00E-05	0.00069884
gene1456	*rsaL*	627.92	209.122	−1.58624	−2.86187	5.00E-05	0.00069884
gene292	*oprE*	20.563	6.97732	−1.5593	−2.60418	5.00E-05	0.00069884
gene4352	*tufB*	29.6318	10.2742	−1.52813	−2.49072	5.00E-05	0.00069884
gene547	PA0542	65.1469	22.9895	−1.50272	−2.22129	0.0011	0.0100167
gene4138	*oprG*	27.1767	9.75134	−1.4787	−2.06861	0.0021	0.015976
gene5233	*glnA*	83.3418	30.4831	−1.45103	−2.97103	5.00E-05	0.00069884
gene3389	*clpP2*	23.5314	8.74695	−1.42774	−1.8175	0.007	0.0372301
gene3722	*frr*	271.131	103.065	−1.39543	−2.83483	5.00E-05	0.00069884
gene308	PA0306a	39.0824	15.5097	−1.33335	−1.82724	0.00395	0.0247286
gene5035	*azu*	166.858	66.2508	−1.33261	−2.52071	5.00E-05	0.00069884
gene2673	*aceA*	17.8349	7.20472	−1.30769	−2.22915	0.00055	0.00541885
gene1560	PA1533	104.495	42.4434	−1.29983	−2.00187	0.00245	0.0179567
gene5680	*atpD*	29.3639	12.0031	−1.29064	−2.40291	5.00E-05	0.00069884
gene5623	*nrdJa*	8.16542	3.36928	−1.27709	−1.95924	0.0016	0.0126526
gene1176	*imm2*	134.36	56.4069	−1.25216	−1.83929	0.0038	0.0247286
gene4588	PA4500	8.97191	3.82949	−1.22826	−1.72289	0.00615	0.0345435
gene833	PA0820	25.8366	11.1948	−1.20659	−1.74814	0.0039	0.0247286
gene1535	*Tli4*	22.776	9.92586	−1.19825	−1.80905	0.00295	0.0206157
gene1457	*lasI*	62.0684	27.0521	−1.19812	−2.10094	0.0005	0.00500833
gene3201	*wbpM*	21.5844	9.88673	−1.12643	−2.13953	0.00015	0.001803
gene3907	PA3836	16.2993	7.47594	−1.12448	−1.65887	0.0072	0.0379579
gene656	*vfr*	25.6241	11.8251	−1.11565	−1.55319	0.0107	0.0494669
gene4399	*mvaT*	1,061.23	491.469	−1.11057	−2.22426	0.00025	0.00268304
gene414	*pilJ*	13.8071	6.57893	−1.06948	−1.91188	0.0015	0.01202
gene3801	PA3732	230.837	111.46	−1.05035	−2.14155	0.0002	0.00231154
gene3598	*bfrB*	3,320.52	1,612.16	−1.04241	−2.13433	0.00035	0.00369035
gene5682	*atpA*	96.9071	47.1674	−1.03881	−2.14129	0.0004	0.00414483
gene1116	*fliD*	13.5333	6.6493	−1.02524	−1.65814	0.0066	0.0357351
gene1976	PA1939	8.32965	4.10377	−1.0213	−1.59073	0.0075	0.0385256
gene523	*nirS*	48.272	24.8277	−0.959236	−1.94776	0.0012	0.0106059
gene5157	*pilO*	108.242	55.8768	−0.953941	−1.64711	0.0042	0.0257571
gene4451	*sodB*	285.132	147.322	−0.952659	−1.96854	0.00115	0.0103157
gene4680	*ccpR*	20.1423	10.4282	−0.949741	−1.57754	0.0077	0.0392178
gene5155	*pilQ*	12.2848	6.43035	−0.9339	−1.68001	0.00445	0.0267445
gene1613	*sdhB*	252.039	134.811	−0.902714	−1.85065	0.00135	0.0114275
gene1179	*nrdB*	63.8121	34.8794	−0.871454	−1.77239	0.0023	0.0172787
gene978	*oprD*	27.184	14.9777	−0.859943	−1.61922	0.0059	0.0337705
gene1175	*pys2*	96.6484	53.9177	−0.841987	−1.70527	0.0024	0.0178074
gene3937	PA3866	57.9267	32.6472	−0.827268	−1.70364	0.00285	0.0201512
gene5362	*hemB*	44.5942	25.3888	−0.812662	−1.57048	0.00735	0.0384117
gene2595	PA2560	11.782	0	-inf	-nan	0.00015	0.001803
gene2851	PA2805	7.48308	0	-inf	-nan	0.00135	0.0114275

**Note:**

-inf, not define; -nan, not available.

**Figure 5:**
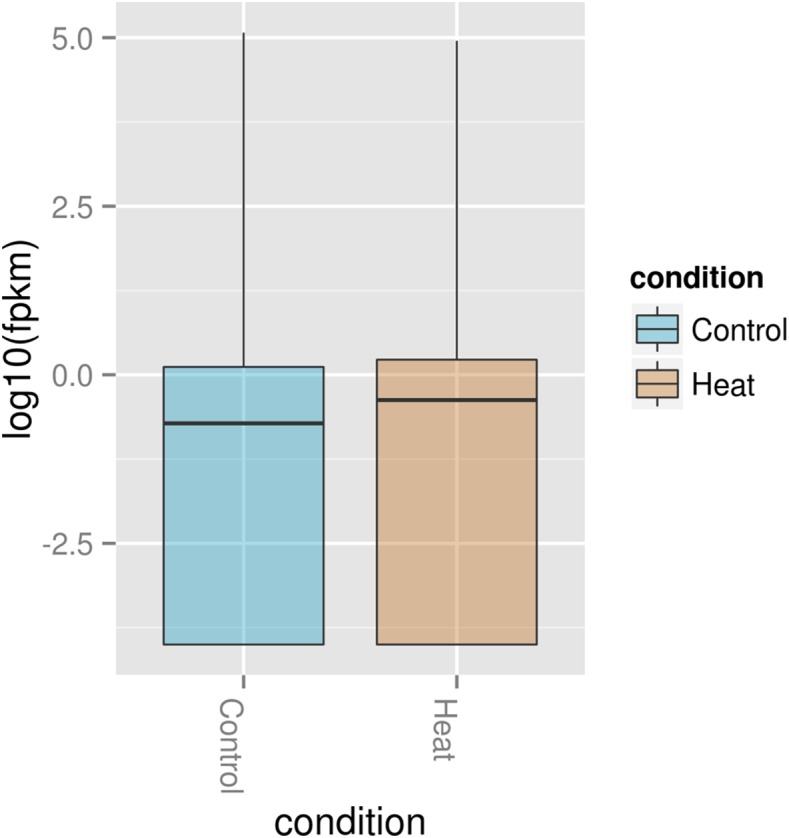
Box plot of normalized expression values of RNA in *P. aeruginosa* PAO1 exposed to 37 and 46 °C. The Box plot shows that how log_2_ transformed values of the expressed genes in *P. aeruginosa* PAO1 when grown at 37 °C (Control) and 46 °C (Heat). A Box plot of gene expression distribution is shown for genes falling into each bin. The bottom and top of the Box are the first and last quartiles and the line within the box is the 50th percentile [the median] of the points in the group. The Box plot median shows that expression at 37 °C (Control) is slightly lower than 46 °C (Heat).

The Box plot (Fig. 5) shows that *P. aeruginosa* PAO1 gene global expression at 37 °C is slightly lower than at 46 °C suggesting that when exposed to elevated temperature. *P. aeruginosa* PAO1 cells respond to heat very quickly (within 30 min). There is a large variance in the response of *P. aeruginosa* PAO1 genes to heat shock at 46 °C, as evidenced by the large scatter of values along the vertical axis in Fig. 5. Nonetheless, the median for gene expression values is still higher in *P. aeruginosa* PAO1 cells subjected to heat shock at 46 °C compared to 37 °C. Our transcriptome analysis therefore shows that several genes in *P. aeruginosa* PAO1 are temperature-dependent (Fig. S1).

### A number of *P. aeruginosa* PAO1 genes are regulated by temperature

Of the 133 differentially expressed genes identified (*q* ≤ 0.05 fixed as the cut-off values), 55 genes were upregulated (Table 3) and 78 genes were downregulated (Table 4). The gene most upregulated under our 30 min heat shock experiment at 46 °C was *clpB. ClpB* is a well-known heat shock gene in bacteria. In our study, its expression was much higher than all other heat shock genes (log_2_ fold change of 4.896). Another highly upregulated gene was one of unknown function. Therefore, its role in heat shock response needs to be further investigated.

**Figure 6:**
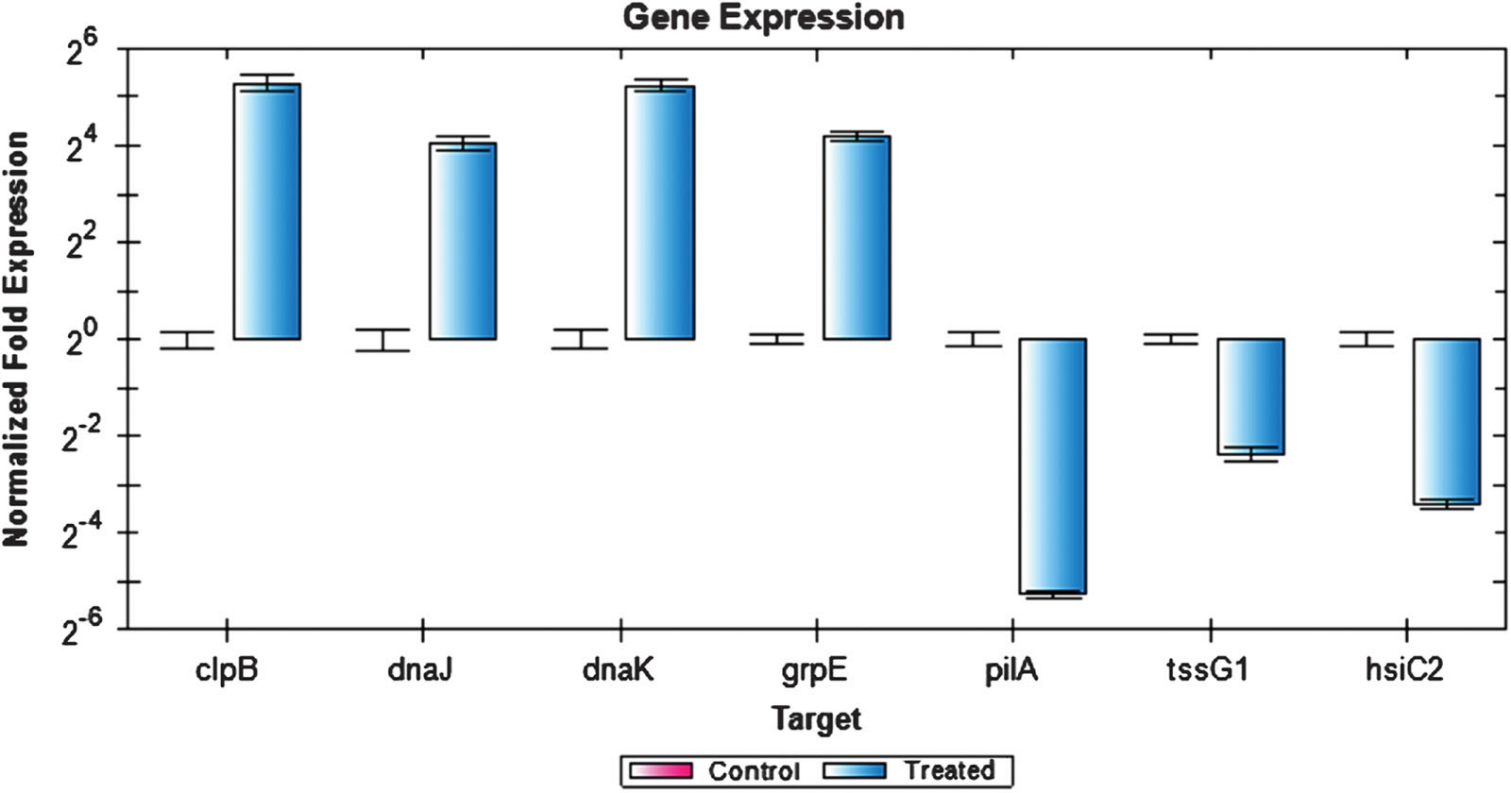
Expression analysis of *P. aeruginosa* PAO1 genes using RT-PCR. The expression values of four upregulated (*clpB, dnaJ, dnaK* and *grpE*) and three downregulated (*pilA, tssG1,* and *hsiC2*) genes as analyzed by RT-PCR. Rreference genes *cheZ, proC, recA, rpoB, gyrB, oprL,* and *plsY,* with an *M* value of less than 0.7 were selected.

Figure 6 depicts the expression of selected genes quantified using RT-PCR. Expression levels of the genes tested were closely correlated with the data obtained from RNA-seq experiment. Notably, *clpB, dnaJ, dnaK,* and *grpE* genes were upregulated while *tssG1, hsiC2,* and *pilA* genes were downregulated. However, the expression values obtained by RT-PCR were slightly higher compared to the RNA-seq expression value. This could be due to the use of gene specific primers in the RT-PCR compared to the RNA-seq, which used universal PCR primers for the cDNA amplification step. Additionally, the samples used for RT-PCR were from an independent heat shock experiment, which may account for the slight variation in the overall gene expression values.

## Discussion

It is anticipated that bacteria are subject to many abiotic changes in both the external environment and in a mammalian host. One of the frequent abiotic stresses that many pathogens such as *P. aeruginosa* PAO1 will encounter is elevated temperature. In order to study the *P. aeruginosa* PAO1 heat shock response, we used RNA-seq coupled with NGS. We performed a global transcriptional analysis of gene expression after exposing this opportunistic pathogen to heat shock.

The gene most upregulated under our 30 min heat shock experiment was *clpB*. It has previously been documented that ClpB plays a vital role in the bacterial heat shock response and adaptation to elevated temperatures, being responsible for breaking down massive bacterial protein aggregates (Schirmer et al., 1996). The levels of ClpB and other chaperones, such as DnaKJ, GrpE, and GroESL, need to be in balance (Kedzierska & Matuszewska, 2001). In this work, *dnaK, dnaJ, groEL* were expressed in that descending order, which is in agreement with the work of Kedzierska & Matuszewska (2001). Another group of genes classified as being involved in damage control and repair were also significantly upregulated. Among these were the genes *groEL*, *grpE* and *hslU* which could potentially provide more protection against the damage caused by heat shock (Mogk et al., 1999; Motohashi et al., 1999; Zolkiewski, 1999).

The second most highly over expressed gene in the heat shock experiment was PA0779 (*asrA* gene). Overexpression of *asrA* has previously been reported to lead to the induction of the heat shock response in *P. aeruginosa* (Kindrachuk et al., 2011). Our transcriptome data are there for comparable to the work of Kindrachuk et al. (2011) from two perspectives: (i) The heat shock genes *htpG*, *groES*, *clpB*, *dnaJ* and *hslV* but not *ibpA* gene were induced by overexpression of *asrA* gene, and (ii) the known heat shock sigma factor rpoH is involved in mediating *P. aeruginosa* stress response to tobramycin and heat shock through asrA. In the transcriptome of *P. aeruginosa* PAO1 cells subject to heat shock we observed the upregulation of rpoH, a well-coordinated phenomenon related to the upregulation of PA0779 (*asrA* gene). It has been reported that the function of AsrA protein goes beyond simply the heat shock response and that it is also a key mediator of tobramycin antibiotic response (Kindrachuk et al., 2011).

Another interesting finding in our *P. aeruginosa* PAO1 heat shock experiments is the upregulation of RsmA (Regulator of secondary metabolism > 2-fold). RsmA or CsrA (carbon storage regulator) is a RNA-binding protein that acts as a posttranscriptional regulatory protein in *P. aeruginosa*. RsmA has been implicated in a number of processes such as the regulation of secondary metabolism, and the expression of several genes related to quorum sensing, motility and virulence determinants (Kay et al., 2006).

In *P. aeruginosa*, RsmA has been shown to negatively control the expression of several virulent genes and quorum sensing (Pessi et al., 2001). RsmA has also been shown to exert positive effects on swarming motility, lipase and rhamnolipid (Heurlier et al., 2004). In our work, the quorum sensing gene *lasI* is significantly downregulated, which is in agreement with the reported work of Pessi et al. (2001). The flagella cap protein gene *fliD* was also downregulated in our study. As flagella is one of the required features for swarming in *P. aeruginosa* (Kohler et al., 2000) it is postulated that motility could be affected by elevated heat.

RsmA coordinates its regulation with a small non-coding regulatory RNA molecule, RsmZ (RsmB) (Timmermans & Van Melderen, 2010). In this work, *rsmZ* was downregulated. It has been reported that both positive and negative effects of RsmA are found to be antagonized by RsmZ (Heurlier et al., 2004). In the light of this finding, it is postulated that several virulence genes can be affected by heat shock. Although the effects of RsmA regulation could be minimised due to the downregulation of *rsmZ*. Also, in *P. aeruginosa*, RsmY but not RsmZ, can be bound and stabilized by the RNA chaperone protein Hfq effectively blocking the action of RsmA (Sorger-Domenigg et al., 2007). In our work, neither RsmY nor Hfq were detected, implying that Hfq is irrelevant in the response of *P. aeruginosa* PAO1 to elevated temperature.

The major structural component of bacterial cells is the cell wall, which provides physical protection against environmental stresses such as heat shock. Lipopolysaccharide biosynthesis is an important endotoxin and key component of the bacterial cell wall and membrane. In our work, another gene significantly upregulated was *waaG,* which is responsible for lipopolysaccharide biosynthesis. Suggesting that when exposed to high temperature, *P. aeruginosa* PAO1 upregulates endotoxin production.

The gene (*wbpD*) responsible for *N*-acetyltransferases which is important for *O*-antigen or *O*-polysaccharide biosynthesis in *P. aeruginosa* PAO1 (Wenzel et al., 2005), and it is this region which confers serum resistance to this pathogen (Rocchetta, Burrows & Lam, 1999). Our data imply that when exposed to high temperature such as fever condition in the host being infected by *P. aeruginosa* PAO1, upregulation of genes important for lipopolysaccharide and *O*-polysaccharide biosynthesis confers endotoxin production and resistant to serum killing, thus providing a means to overcome the host defence. This result also suggests a molecular sensing and response of *P. aeruginosa* PAO1 on the changes from the environment to the mammalian host and fever condition.

One of the major effects of heat shock in bacteria is a loss of genome integrity. Three major processes, namely DNA replication, DNA recombination, and DNA repair can help to combat this (Klein & Kreuzer, 2002) but these processes can also be affected by heat changes (López-García & Forterre, 2000). Our work showed the upregulation of *dnaG*, a primase that synthesizes a primer that is essential for DNA replication. We postulate that during heat shock, DNA replication is upregulated, facilitated in part by the *dnaG* gene. The increase in DNA content helping to maintain genome stability, fidelity and integrity under high heat in this pathogen.

We also found that the RNA polymerase core enzyme gene *rpoD* was upregulated during heat shock. This is in agreement with previously reported work of Aramaki & Fujita (1999). The expression of *rpoD*, together with other sigma factors such as *rpoH* and *rpoB* have been well-documented for their role in the fitness of species such as *E. coli* (Barrick et al., 2010) and *P. aeruginosa* (MacLean & Buckling, 2009). The upregulation of *rpoB* in our work leads us to speculate that this will increase survival fitness enabling *P. aeruginosa* PAO1 to withstand the deleterious effect of exposure to increased temperature.

The majority of the significantly downregulated genes identified in this study are of unknown functions and will require further analysis. However, some of the top downregulated genes were discovered to be part of the *P. aeruginosa* type VI secretion system (T6SS). Secretion systems in many prokaryotes vary in terms of their complexity, however these systems incorporate the usage of a single polypeptide to build their path across bacterial cell envelopes (Filloux, Hachani & Bleves, 2008). We therefore speculate that T6SS is involved in interactions with eukaryotic cells possibly contributing to bacterial pathogenesis. However, the precise roles of the proteins produced by the genes in this system are currently unknown. Two genes belonging to this system *tssG1* and *hsiC2* were under-expressed. Down regulation of these genes may help *P. aeruginosa* PAO1 to conserve energy enabling them to produce proteins that protect them at such elevated temperature.

Surprisingly, amongst the genes significantly downregulated in *P. aeruginosa* PAO1, are those of type IV pili genes. *pilA, pilB, pilJ, pilO, pilQ* (in ascending order) were all downregulated. *P. aeruginosa* expresses polar type IV pili for adhesion to various materials and twitching motility (Chiang, Habash & Burrows, 2005). It has been reported that in dispersed *P. aeruginosa* PAO1 under nutrient-limiting conditions, biofilm dispersion is associated with a decreased expression of pilus (*pilA*) genes cells (Sauer et al., 2004). In light of this, it appears that biofilm dispersion could be affected by elevated heat, as judged by the reduction in the expression of a myriad of type IV pili genes. In order to avoid the deleterious effect of heat shock *P. aeruginosa* PAO1 may disperse from the static biofilm and convert to planktonic cells which are free to escape the heated site.

To conclude, heat shock has a profound impact in the opportunistic pathogen *P. aeruginosa* PAO1. It affects a number of key genes related to a number of processes. Chief amongst these are chaperones, heat shock proteins, proteases, heat shock-related sigma factor, and posttranscriptional regulatory proteins (regulating secondary metabolism and the expression of virulence). In addition, genes involved in regulating quorum sensing, motility and membrane and pili and biofilm formation are also differentially regulated by heat shock. We believe that transcriptome analysis using RNA-seq technology can be a very useful approach to study gene expression profiling and further understand the mechanism employed by pathogens, especially in establishing an infection.

## Supplemental Information

10.7717/peerj.2223/supp-1 Supplemental Information 1 Heat map of P. aeruginosa PAO1 genes expression of all regulated genes at 37 and 46 °C. Overview of genes heat map profile at alpha = 0.05 significant level.

10.7717/peerj.2223/supp-2 Supplemental Information 2 Supplementary Data 1.

## Additional Information and Declarations

### Data Deposition

The following information was supplied regarding data availability: The DNA sequences from this transcriptomics project are deposited at Sequence Read Archive (NCBI/SRA) under the accession number SRP066875. The transcriptome data are deposited in BioProject in GenBank via Bioproject number PRJNA304652 accessible at: http://www.ncbi.nlm.nih.gov/bioproject/?term=PRJNA304652.
