# Supplementary figures and images for: Transcriptome analysis of Pseudomonas aeruginosa PAO1 grown at both body and elevated temperatures
Source: PeerJ. 2016 Jul 19;4:e2223. doi: 10.7717/peerj.2223 (PMC4957987; doi:10.7717/peerj.2223)

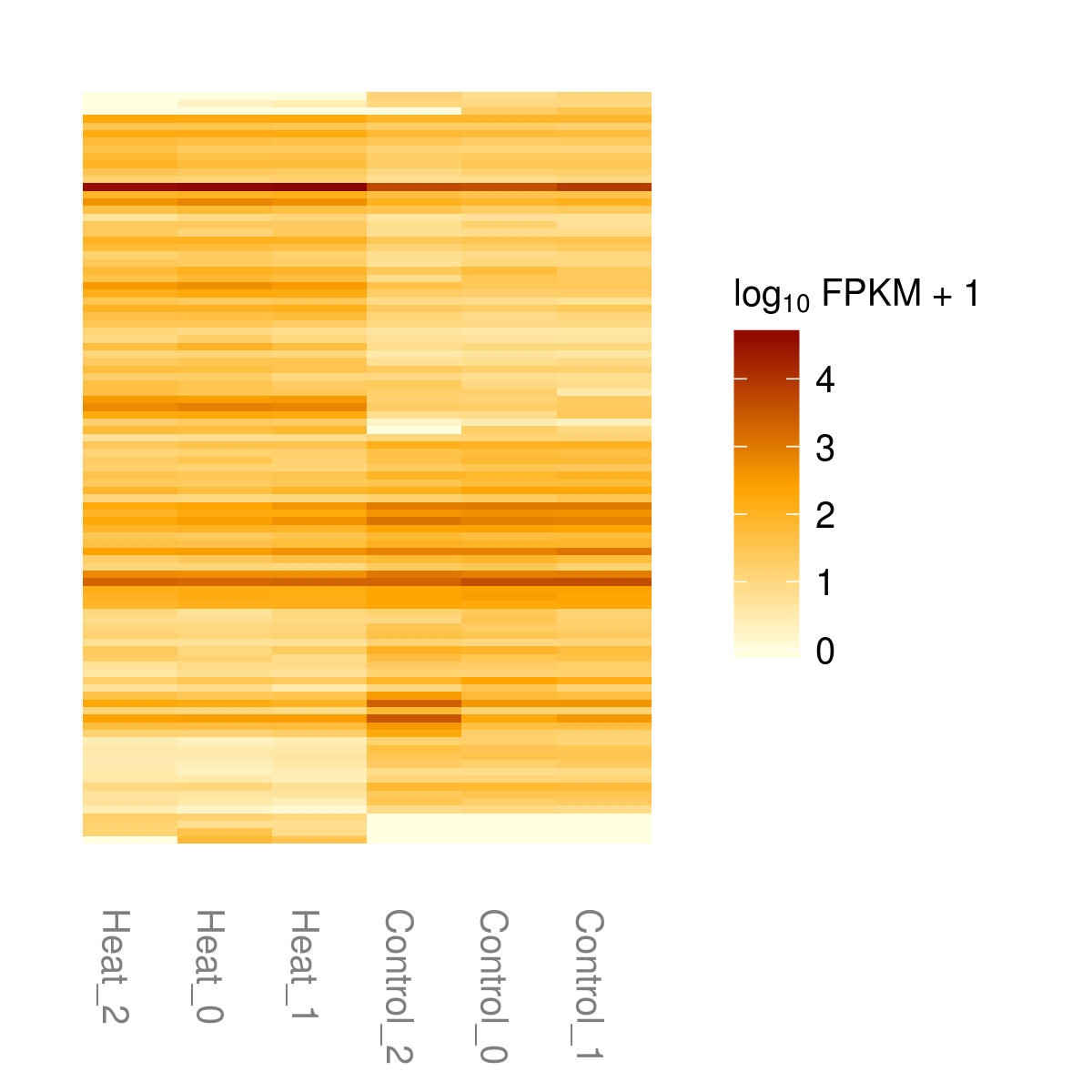

Supplement: Supplemental Information 1 — Overview of genes heat map profile at alpha = 0.05 significant level. [file peerj-04-2223-s001.png]
